# Minichromosome maintenance gene family: potential therapeutic targets and prognostic biomarkers for lung squamous cell carcinoma

**DOI:** 10.18632/aging.204399

**Published:** 2022-11-28

**Authors:** Xuejie Yang, Chunrong Wang, Hui Nie, Jianhua Zhou, Xiaoyun He, Chunlin Ou

**Affiliations:** 1 Department of Pathology, Xiangya Hospital, Central South University, Changsha 410008, Hunan, China; 2 Departments of Ultrasound Imaging, Xiangya Hospital, Central South University, Changsha 410008, Hunan, China; 3 National Clinical Research Center for Geriatric Disorders, Xiangya Hospital, Central South University, Changsha 410008, Hunan, China

**Keywords:** MCM family, lung squamous cell carcinoma, prognosis, methylation, immune cells

## Abstract

The minichromosome maintenance (MCM) gene family comprises of ten members with key roles in eukaryotic DNA replication and are associated with the occurrence and progression of many tumors. However, whether the MCM family contributes to lung squamous cell carcinoma (LUSC) is unclear. In this study, we performed bioinformatic analysis to identify the roles of MCM genes in patients with LUSC. We also evaluated their differential gene expression, prognostic correlation, DNA methylation, functional enrichment of genetic alterations, and immunomodulation. According to the Tumor Immune Estimation Resource database, the expression of MCM2-10 mRNA was elevated in LUSC tissues. According to the Gene Expression Profiling Interactive Analysis database, MCM2–8 and MCM10 were considerably upregulated in LUSC tissues, and protein levels of all MCMs were increased in LUSC tissues. In addition, among the MCM family members, the expression of MCM3 and MCM7 showed the strongest correlation with the prognoses of patients with LUSC. To clarify the role and mechanisms of the MCM family, Kyoto Encyclopedia of Genes and Genomes and Gene Ontology enrichment studies were performed. We detected a significant correlation between the expression patterns of MCM family members and infiltrating immune cells. In conclusion, our results improve the understanding of the aberrant expression of MCM family members in LUSC. These findings demonstrate the potential of the MCM family as therapeutic targets and biomarkers for the diagnosis and prognosis of LUSC.

## INTRODUCTION

Lung cancer accounts for 11.4% of all cancer cases worldwide, resulting in tremendous financial and medical burdens on the society each year [1]. Depending on the age of the patient or degree of tobacco exposure, lung squamous cell carcinoma (LUSC), a subtype of non-small cell lung cancer, comprises around 40% of all lung cancer cases. Currently, surgical resection, chemotherapy, radiation, targeted treatment, and immunotherapy are being used to treat lung cancer. LUSC has a poor clinical prognosis compared to lung adenocarcinoma, and only a few molecular targeted treatments are available [2, 3]. Additionally, crucial biomarkers and specific targets for LUSC prognosis remain unidentified, making it imperative to find new biomarkers and targeted medicines for improving therapeutic outcomes [4, 5].

Ten members of the MCM family, serum response factor (SRF, also known as MCM1) and MCM2–10, were initially found in *Saccharomyces cerevisiae* [6]. The MADS-box family of transcription factors were defined based on the primary sequence similarity among numerous proteins from a diverse range of eukaryotic organisms including yeasts, plants, insects, amphibians, and mammals [7]. By controlling the expression of cell division cycle 6 and MCM2–7 genes, MCM1, an ancient and evolutionarily conserved transcription factor in the MADS-box family, indirectly affects DNA replication [8, 9]. The MCM2–7 proteins are heterohexameric complexes that serve as primary helicases to unwind the helical structure of DNA by acting on the origins of replication [10]. MCM8 and MCM9 function as hexameric ATPase/helicase complexes that may be involved in homologous recombination repair induced by interstrand crosslinks [11]. Additionally, MCMs show potential as diagnostic and prognostic indicators, as they are overexpressed in various cancer tissues and carcinoma cell lines [12–14]. Although potential biomarkers and genes associated with LUSC are being widely examined [15, 16], the prognostic importance of the MCM family in the development of LUSC remains unclear. In this study, we used research databases and bioinformatic tools to assess the expression of MCMs in LUSC and analyze their prognostic value, which could be useful for improving treatment outcomes.

## RESULTS

### Aberrant expression of MCM family members in LUSC

The Tumor Immune Estimation Resource (TIMER) database was used to examine transcriptional levels of MCMs and to compare their expression in diverse cancer types to those in healthy tissues. The mRNA expression of MCM2–10 was substantially higher in LUSC tissues than in healthy tissues, whereas MCM1 levels showed no significant difference (Figure 1A–1J). Gene Expression Profiling Interactive Analysis (GEPIA2), which contains resources distinct from those in the TIMER database, was used to further explore the mRNA expression of the MCM family genes. We found that all MCM factors, except for MCM1 and MCM9, exhibited considerably higher expression in LUSC than in normal tissues (*P* < 0.05) (Figure 1K).

**Figure 1:**
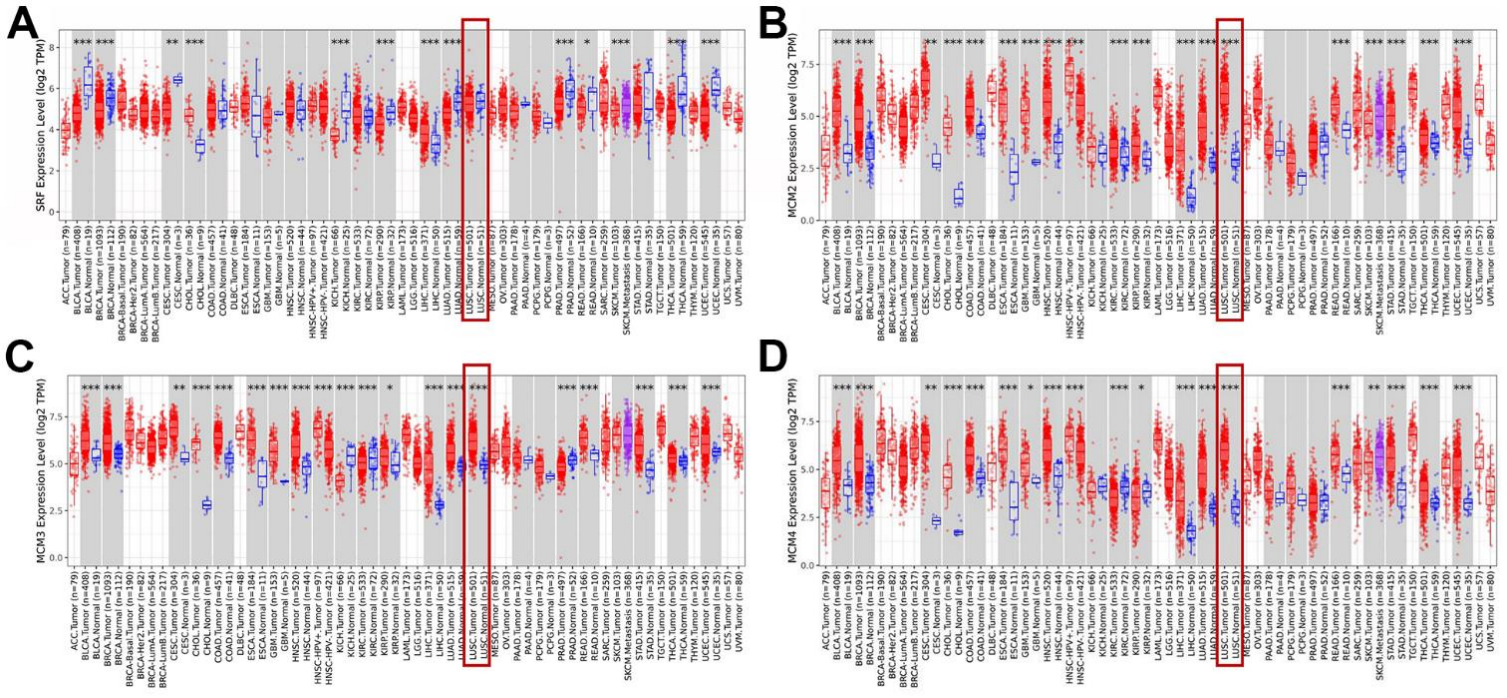
**Expression of minichromosome maintenance (MCM) family members in lung squamous cell carcinoma (LUSC).** (**A**–**D**) Expression of MCM1–10 in pan-cancer. *p < 0.05, **p < 0.01, ***p < 0.001 compared with control.

**Figure 1:**
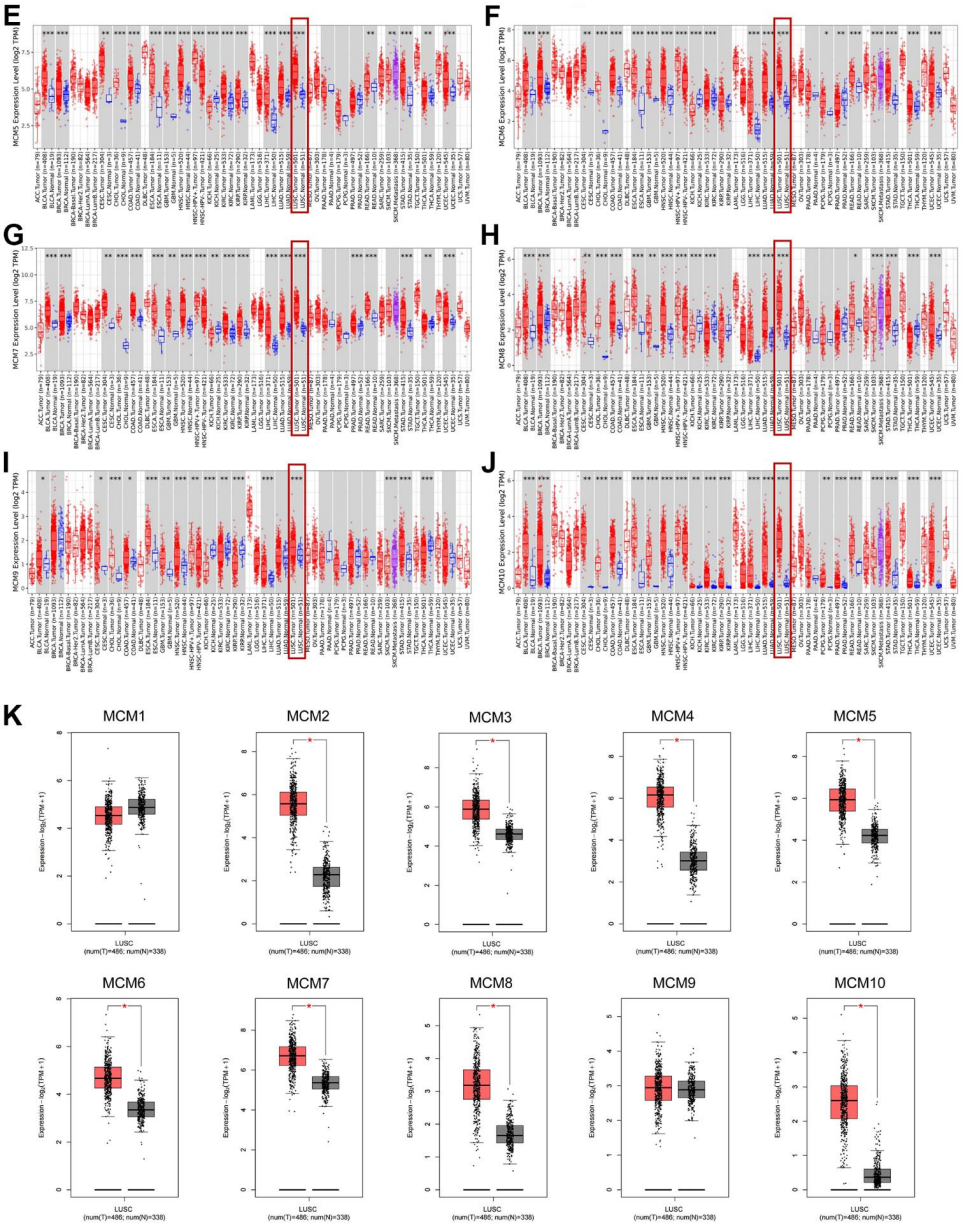
**Expression of minichromosome maintenance (MCM) family members in lung squamous cell carcinoma (LUSC).** (**E**–**J**) Expression of MCM1–10 in pan-cancer. (**K**) Expression of MCMs in LUSC. *p < 0.05, **p < 0.01, ***p < 0.001 compared with control.

**Figure 2:**
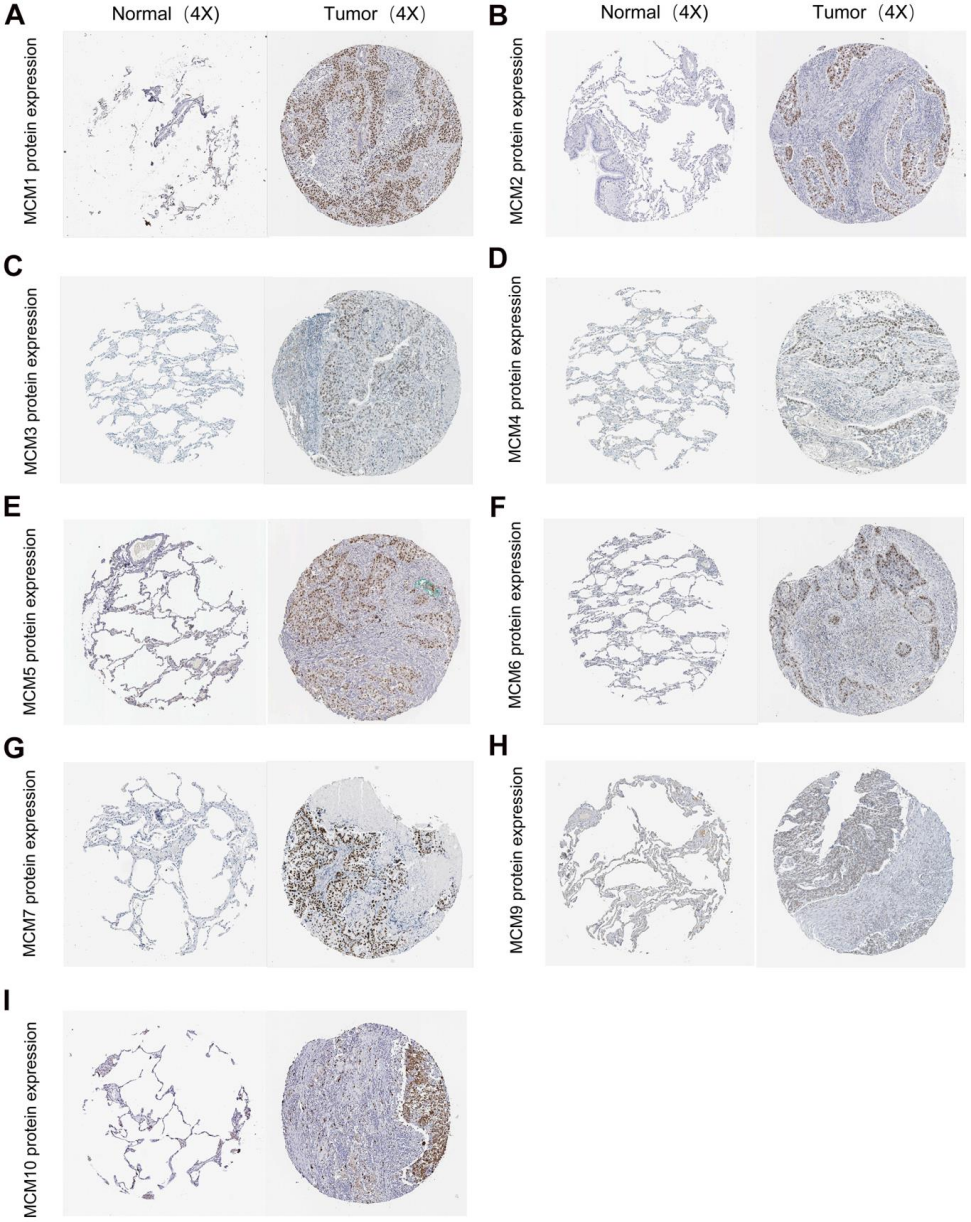
**Typical immunohistochemistry images of minichromosome maintenance (MCM) family members.** (**A**–**I**) Comparison of the expression of MCM1–10 in lung squamous cell carcinoma (LUSC) tissues with those in non-cancerous tissue (100×) using the data from the Human Protein Atlas database.

We further investigated the results of immunohistochemical staining of MCM family members using data from the Human Protein Atlas database to evaluate the protein levels of MCMs in LUSC. Figure 2 shows that LUSC tissues expressed higher protein levels of MCM3/4/5, compared to the normal lung tissues (not detected versus medium, respectively) (Figure 2C–2E). Similar findings were observed for the protein expression levels of MCM2/6/7/10 (not detected versus high, medium versus high, not detected versus high, and low versus medium, respectively) (Figure 2F, 2I). These findings are consistent with our previous findings regarding the mRNA expression of the MCM family genes. Moreover, MCM1 was not expressed in normal lung tissues and showed medium expression in LUSC tissues (Figure 2A), whereas MCM9 showed low expression in normal tissues and medium expression in LUSC tissues (Figure 2H).

### Correlation between MCM mRNA expression and clinicopathological parameters in patients with LUSC

**Figure 3:**
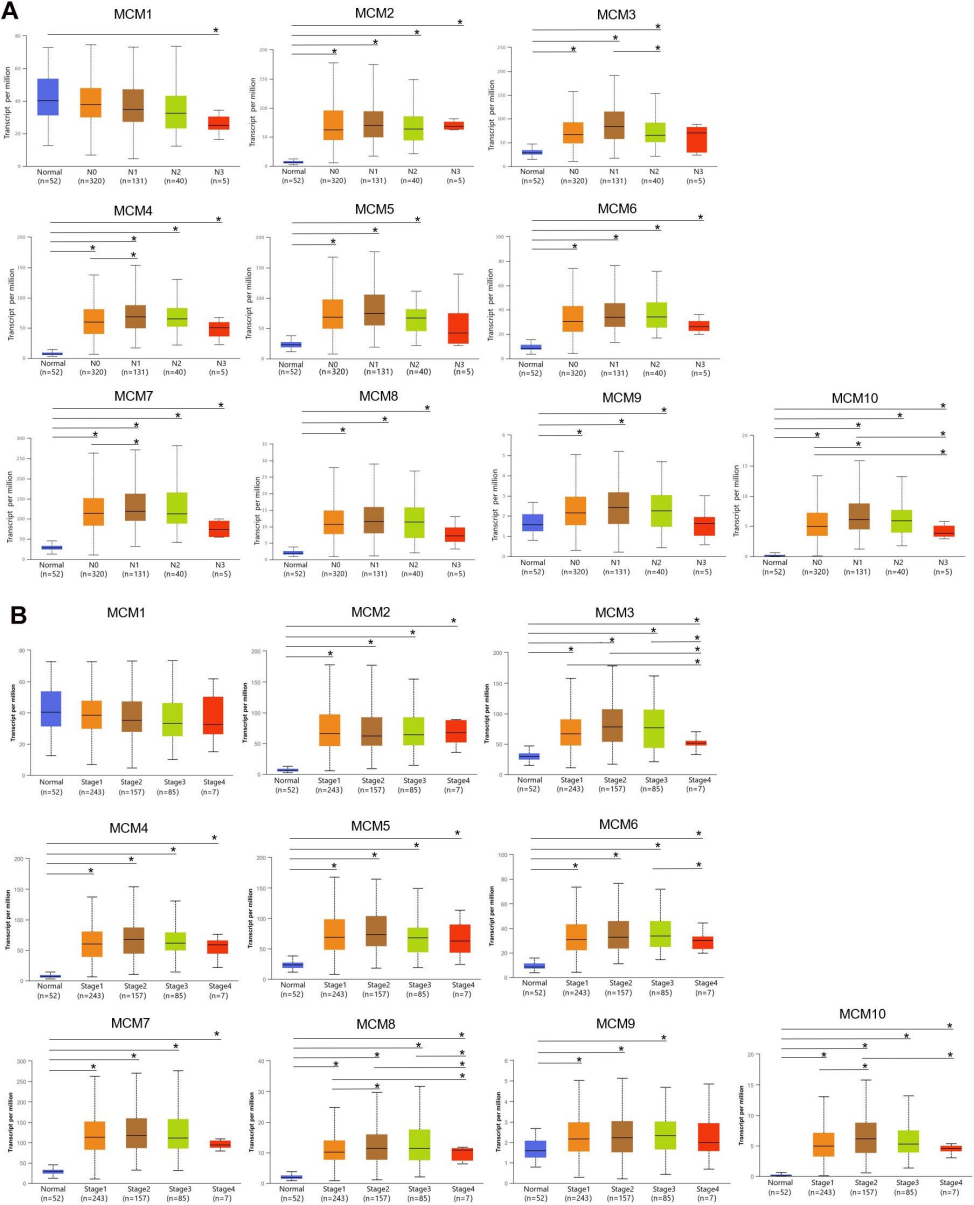
**Correlation between clinical pathology and minichromosome maintenance (MCM) mRNA expression determined using UALCAN.** (**A**) Association of lymph node metastases in patients with lung squamous cell carcinoma (LUSC) with MCM family mRNA expression. (**B**) Association of pathological stage of patients with LUSC with MCM family mRNA expression. *p < 0.05.

The association between the mRNA expression of the ten MCM genes and lymph node metastases in LUSC was examined using the UALCAN database. At all stages of lymph node metastasis, the MCM2/4/6/7/10 mRNA expression in LUSC tissues were higher than those in normal tissues. Additionally, the mRNA expression of MCM3/5/8/9 tended to be higher in tumors with N0–N2 stage lymph node metastasis. At the N0–N3 stage of lymph node metastasis, MCM4/6/7/10 showed the lowest expression in N3 tumors, which significantly correlated with patient prognosis. In contrast, the expression of MCM2 was highest at N3 stage (Figure 3A). Therefore, the mRNA expression of MCM family members were significantly correlated with individual cancer stages of LUSC. The expression of eight MCM genes (excluding MCM1 and MCM9) was significantly higher in the tumor stage 1–4 subgroups than that in normal lung tissues. However, the expression of MCM9 did not differ between stage 4 LUSC tissue and normal lung tissue. In addition, MCM3/6/8/10 mRNA expression was lower in tumor stage 4 than in tumor stages 1 and 3. These findings suggest that MCMs contribute to the development of LUSC (Figure 3B).

### Prognostic value of MCM gene family in patients with LUSC

**Figure 4:**
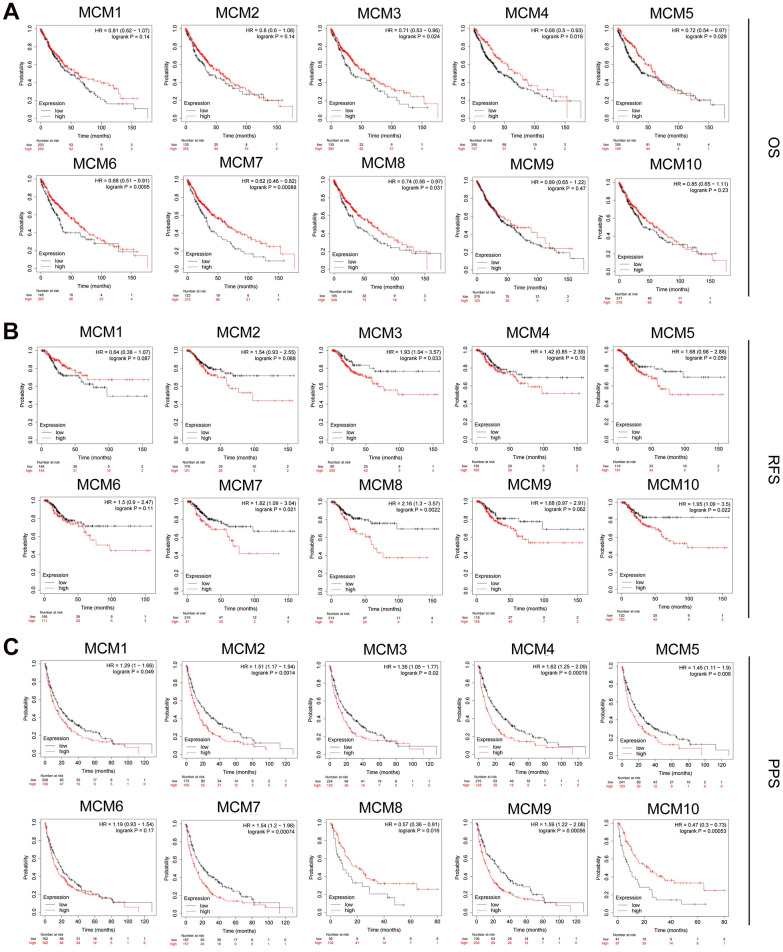
**Prognostic value of MCM family members in lung squamous cell carcinoma (LUSC).** (**A**–**C**) Association of mRNA expression of MCM family members with overall survival (OS), progression-free survival (RFS), and post-progression survival (PPS) in LUSC using Kaplan–Meier plotter database.

Based on mRNA expression in patients with LUSC, the prognostic value of the MCM family was assessed using the Kaplan–Meier plotter database. The clinical prognosis was determined through survival analysis considering post-progression survival (PPS), recurrence-free survival (RFS), and overall survival (OS). We found that higher mRNA expression of MCM3 [OS: hazard ratio (HR) = 0.71 (0.53–0.96), *P* = 0.024], MCM4 [OS: HR = 0.68 (0.5–0.93), *P* = 0.016], MCM5 [OS: HR = 0.72 (0.54–0.97), *P* = 0.029], MCM6 [OS: HR = 0.68 (0.51–0.91), *P* = 0.0095], MCM7 [OS: HR = 0.62 (0.46–0.82), *P* < 0.001], and MCM8 [OS: HR = 0.74 (0.56–0.97), *P* = 0.031] was associated with favorable OS in patients with LUSC and may be suitable targets for improving patient survival and prognosis (Figure 4A). Moreover, increased expression of MCM3 [RFS: HR = 1.93 (1.04–3.75), *P*= 0.033], MCM7 [RFS: HR = 1.82 (1.09–3.04), *P* = 0.021], MCM8 [RFS: HR = 2.16 (1.3–3.57), *P* = 0.002], and MCM10 [RFS: HR = 1.95 (1.09–3.5), *P* = 0.022] was significantly associated with poorer RFS in patients with LUSC (Figure 4B). Upregulation of MCM1 [PPS: HR = 1.29 (1–1.66), *P* = 0.049], MCM2 [PPS: HR = 1.51 (1.17–1.94), *P* = 0.001], MCM3 [PPS: HR = 1.36 (1.05–1.77), *P* = 0.020], MCM4 [PPS: HR = 1.62 (1.25–2.09), *P* < 0.001], MCM5 [PPS: HR = 1.45 (1.11–1.9), *P* = 0.007], MCM7 [PPS: HR = 1.54 (1.2–1.98), *P* < 0.001], and MCM9 [PPS: HR = 1.59 (1.22–2.08), *P* < 0.001] was significantly associated with longer PPS (Figure 4C). The mRNA expression of other MCMs had no significant effect on OS, RFS, or PPS in patients with LUSC (Figure 4). Members of the MCMs family had inconsistent results for OS, RFS, and PPS in patients with LUSC. This situation may be due to the small number of patient samples, and further studies are needed. Among the MCMs, MCM3 and MCM7 showed the most significant correlation with the clinical prognosis of LUSC, indicating the potential of MCM3 and MCM7 as prognostic markers for LUSC.

We further analyzed the prognostic value of the MCM family members at different clinical stages of LUSC using Kaplan–Meier plotter database (Table 1). Overexpression of MCM7 [HR = 2.44 (1.05–5.68), *P* = 0.033] and MCM10 [HR = 2.94 (1.17–7.37), *P* = 0.016] was significantly associated with a poorer RFS in patients with stage I LUSC. The mRNA expression of MCM2 [HR = 7.58 (1.61–35.66), *P* = 0.0028], MCM3 [*P* = 0.038], MCM4 [HR = 4.12 (1.2–14.1), *P* = 0.015], MCM5 [HR = 4.81 (1.04–22.32), *P* = 0.027), MCM8 (HR = 3.63 (1.09–12.09), *P* = 0.024], and MCM9 [HR = 6.2 (0.8–48.12), *P* = 0.046] was significantly associated with poorer RFS in patients with stage III LUSC. These results indicate that some MCM family members can be used as prognostic factors in LUSC, particularly for RFS prediction in patients diagnosed at an advanced stage of LUSC.

**Table 1:** Kaplan–Meier plotter database was used to analyze the prognostic value of MCM family members in different clinical stages of LUSC.

	**Stage1**			**Stage2**			**Stage3**	
	**HR**	***P***		**HR**	***P***		**HR**	***P***
**MCM1**	0.60 (0.27-1.35)	0.220		0.37(0.14-1.01)	**0.043**		2.48(0.74-8.30)	0.130
**MCM2**	1.87(0.78-4.49)	0.150		0.33(0.14-0.76)	**0.006**		7.58(1.61-35.66)	**0.003**
**MCM3**	0.55(0.21-1.47)	0.230		1.67(0.56-4.94)	0.350		——	**0.038**
**MCM4**	2.83(0.85-9.48)	0.077		0.45(0.19-1.04)	0.054		4.12(1.20-14.10)	**0.015**
**MCM5**	2.03(0.92-4.50)	0.075		0.50(0.21-1.18)	0.110		4.81(1.04-22.32)	**0.027**
**MCM6**	2.19(0.94-5.10)	0.064		0.38(0.16-0.87)	**0.018**		4.57(0.57-36.66)	0.120
**MCM7**	2.44(1.05-5.68)	**0.033**		0.38(0.17-0.89)	**0.021**		5.83(0.74-46.21)	0.061
**MCM8**	1.95(0.88-4.33)	0.093		0.58(0.24-1.40)	0.220		3.63(1.09-12.09)	**0.024**
**MCM9**	1.86(0.79-4.34)	0.150		0.56(0.21-1.50)	0.240		6.20(0.80-48.12)	**0.046**
**MCM10**	2.94(1.17-7.37)	**0.016**		0.61(0.23-1.65)	0.320		2. 02(0.43-9.59)	0.370

Bold font indicates significant difference.

### Genetic alterations and functional enrichment analysis of MCM family members

**Figure 5:**
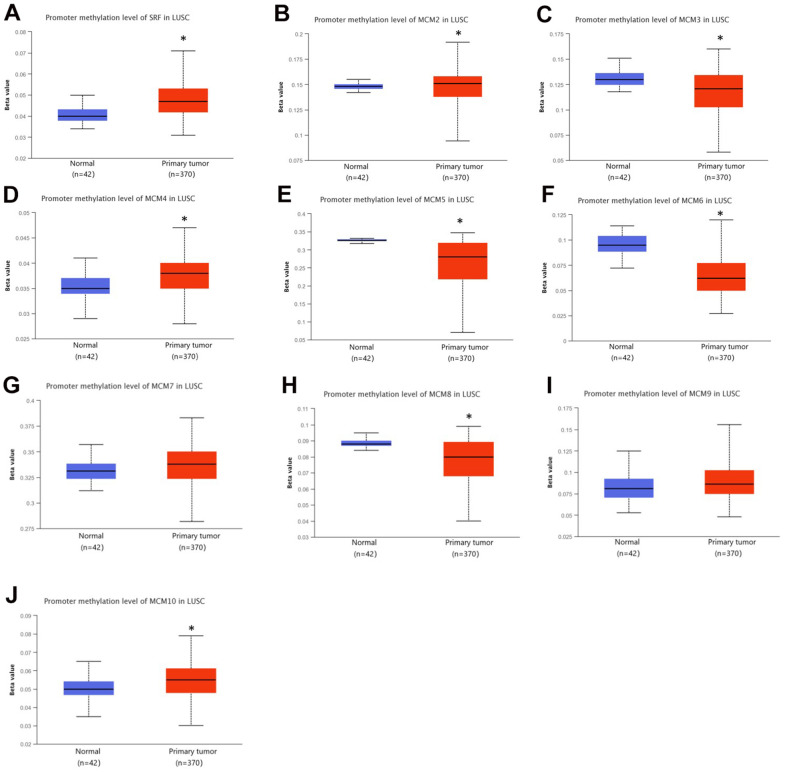
**DNA methylation levels of minichromosome maintenance (MCM) family genes in lung squamous cell carcinoma (LUSC).** (**A**–**J**) DNA methylation change of MCM1–10 in LUSC investigated using the UALCAN database. *p < 0.05 compared with control.

Genetic alterations are well-known to be major factors influencing cancer development. We determined the methylation levels of MCM genes in patients with LUSC using the UALCAN database. The DNA methylation levels of MCM3/5/6/8 were significantly lower in LUSC samples than in normal tissues, whereas those of MCM1/2/4/7/10 were significantly higher. Other MCM family members showed no significant differences between normal and cancer tissues (Figure 5). Variations in the methylation levels of MCM genes may have been caused by differences in their mRNA expression. Thus, DNA methylation-targeting drugs may be useful for treating cancer.

**Figure 6:**
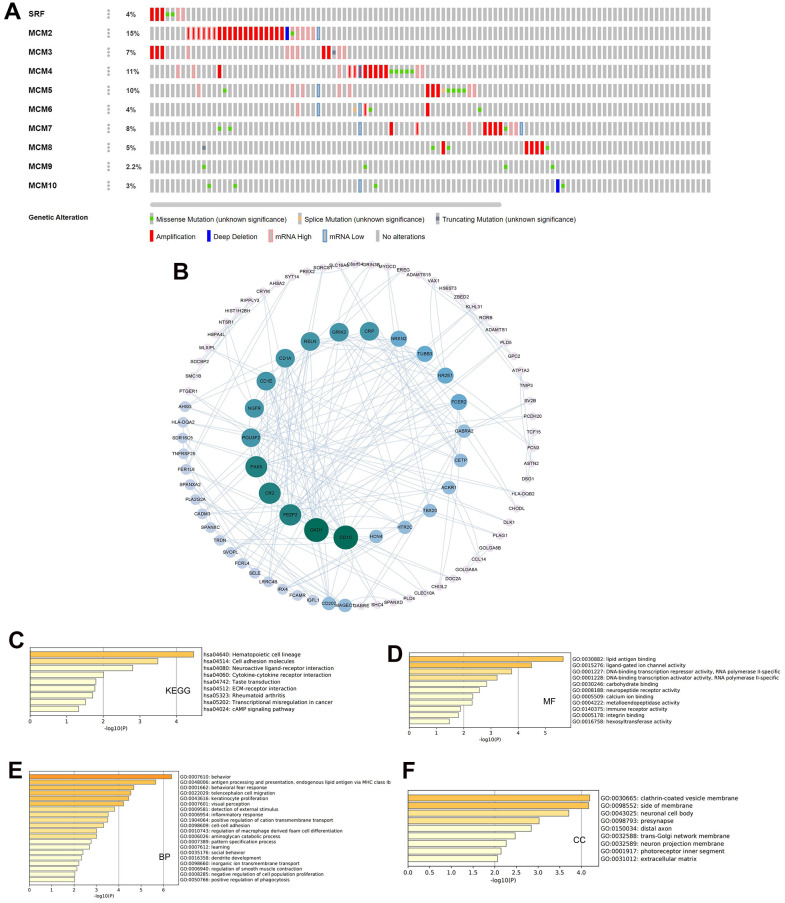
**Genetic alterations and pathway enrichment analysis of minichromosome maintenance (MCM) family in LUSC.** (**A**) Summary of mutation rates in each MCM member in LUSC. (**B**) Protein–protein interaction (PPI) network of the interaction partners of MCM family members built using cBioPortal and Cytoscape. (**C**–**F**) KEGG enrichment pathway analysis of molecular functions, biological processes, and cellular components of co-expressed genes.

In addition, we used the cBioPortal database to conduct a series of studies to verify the status of genetic alterations in the MCM family. We found that 80 of 178 samples (45%) contained alterations in MCM genes, with MCM2 showing the largest proportion of alterations (15%). The two most prevalent genetic changes in the MCM family were mRNA upregulation and amplification (Figure 6A). Next, we identified co-expressed genes with threshold values of |log2 fold-change| ≥ 0.45 and *P* < 0.05 using the cBioPortal database (Supplementary Table 1) and used Cytoscape v.3.9.0 to generate a map of the co-expression networks of key genes related to the MCM family (Figure 6B).

The Metascape database was utilized for Gene Ontology (GO) and Kyoto Encyclopedia of Genes and Genomes (KEGG) pathway analysis of co-expressed genes. Hematopoietic cell lineage, cell adhesion molecules, neuroactive ligand-receptor interaction, and cytokine–cytokine receptor interaction were explored for co-expressed genes using KEGG pathway analysis (Figure 6C). Molecular function analysis indicated that the genes were primarily involved in lipid antigen binding, ligand-gated ion channel activity, and immune receptor activity (Figure 6D). Biological process analysis indicated that these genes were mainly involved in antigen processing and presentation and behavioral fear responses (Figure 6E). Cellular component analysis revealed that these genes were frequently associated with clathrin-coated vesicle membranes and neuronal cell bodies (Figure 6F). These results indicate that the MCM family is involved in antigen processing and presentation, immune receptor activity, and cytokine-cytokine receptor interaction in LUSC, which may impact immune infiltration of the tumor microenvironment (TME) in LUSC tissues.

### Association of expression of MCM family members with immune infiltration in LUSC

The correlation between the MCM gene family and immune cell infiltration was investigated using the TIMER database. The results showed that expression of MCM1 mRNA was significantly associated with infiltration of CD8+ T cells [correlation coefficient (cor) = −0.205, *P* < 0.05] and CD4+ T cells (cor = 0.260, *P* < 0.05). Expression of MCM2 was significantly associated with B-cell (cor = 0.112, *P* < 0.05) and macrophage (cor = − 0.978, *P* < 0.05) infiltration, while MCM3 expression was significantly associated with infiltration of CD4+ T cells (cor = 0.178, *P* < 0.05) and macrophages (cor = −0.176, *P* < 0.05). Additionally, MCM4 expression was significantly associated with infiltration of CD4+ T cells (cor = 0.143, *P* < 0.05) and macrophages (cor = −0.093, *P* < 0.05), while expression of MCM5 was significantly associated with infiltration of CD8+ T cells (cor = −0.143, *P* < 0.05), CD4+ T cells (cor = 0.152, *P* < 0.05), and macrophages (cor = −0.110, *P* < 0.05). MCM7 expression was significantly associated with macrophage infiltration (cor = −0.179, *P* < 0.05), and expression of MCM8 was significantly associated with infiltration of CD4+ T cells (cor = 0.139, *P* < 0.05), while MCM9 expression was significantly associated with infiltration of CD4+ T cells (cor = 0.203, *P* < 0.05). Finally, MCM10 expression was significantly associated with macrophage infiltration (cor = −0.185, *P* < 0.05) (Figure 7). These results suggest that MCM family members affect the immune response in the TME of LUSC.

**Figure 7:**
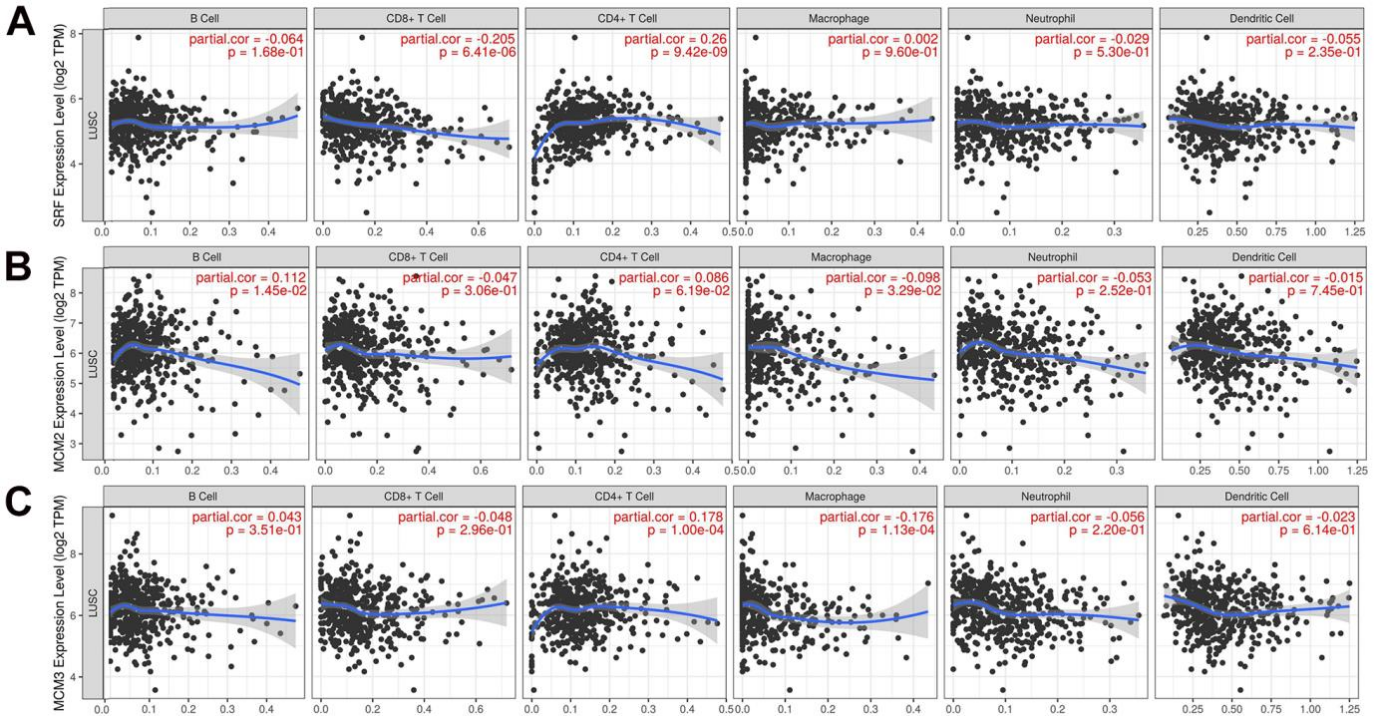
**Relationship between mRNA expression of minichromosome maintenance (MCM) family and immune cell infiltration.** (**A**–**C**) Analysis of association of MCM1–10 mRNA expression with the level of immune cell infiltration in lung squamous cell carcinoma (LUSC) using TIMER database.

**Figure 7:**
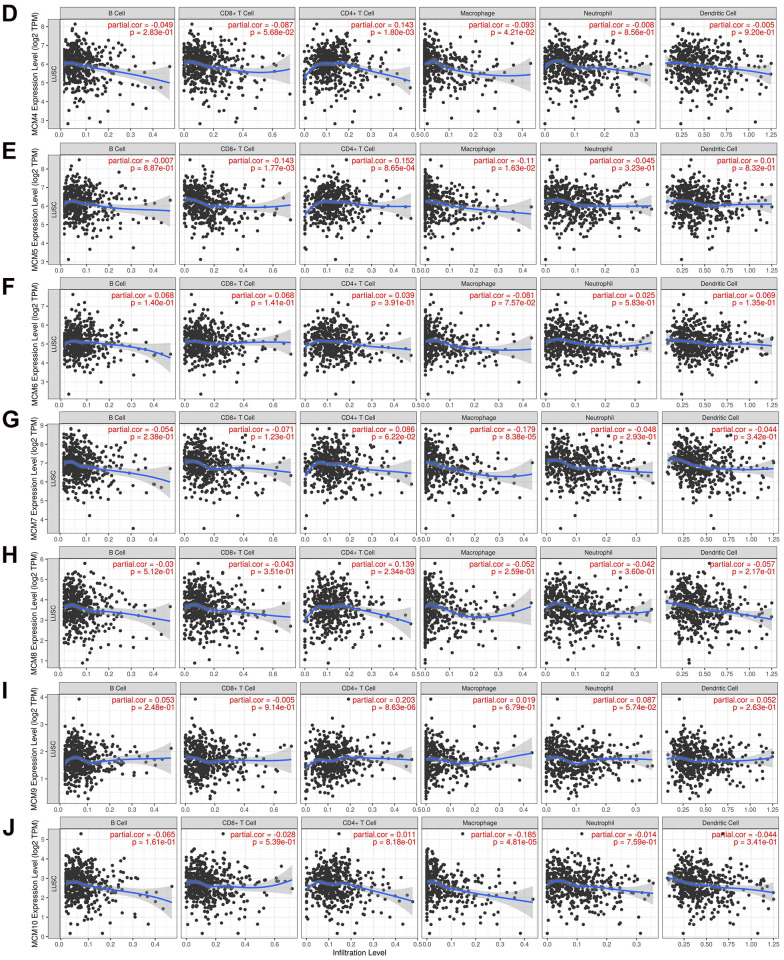
**Relationship between mRNA expression of minichromosome maintenance (MCM) family and immune cell infiltration.** (**D**–**J**) Analysis of association of MCM1–10 mRNA expression with the level of immune cell infiltration in lung squamous cell carcinoma (LUSC) using TIMER database.

Analysis using the TIMER database showed that expression of the MCMs was highly correlated with the signature marker genes of various immune cells in LUSC (Table 2). Notably, MCM1 levels were significantly associated with CD8+ T cells, T cells, M1 and M2 macrophages, T follicular helper (Tfh) cells, Th17 cells, and neutrophils. Expression of MCM2 was significantly correlated with tumor-associated macrophages (TAMs), Th17 cells, and regulatory T cells (Tregs). MCM3 expression was significantly associated with TAMs, M1 macrophages, Tfh, and Th17 cells in LUSC, while MCM4 expression exhibited a strong correlation with B cells, T cells, TAMs, M1 macrophages, dendritic cells, Tfh cells, and Th17 cells. MCM5 expression was significantly associated with most signature marker genes of T cells, TAMs, M1 macrophages, Tfh cells, and Th17 cells. Furthermore, MCM6 expression was associated with B cells, M1 macrophages, Tfh cells, and Th17 cells. MCM7 expression showed high correlations with B cells, T cells, TAMs, M1 macrophages, Tfh cells, and Th17 cells. The mRNA expression of MCM8 was strongly associated with CD8+ T cells, B cells, T cells, TAMs, Th2 cells, Tfh cells, and Th17 cells. The mRNA expression of MCM9 was correlated with M1 macrophages, Tfh cells, Th17 cells, and Tregs. Moreover, B cells, T cells, TAMs, M1 and M2 macrophages, neutrophils, dendritic cells, Th2 cells, and Tfh cells showed favorable correlations with MCM10 expression. Taken together, these findings reveal important relationships between MCM family members and immune-infiltrating cells in LUSC and suggest that these proteins play important roles in the TME of LUSC.

**Table 2:** The correlations between the expression of MCM family members and markers of immune cells.

		**MCM1**		**MCM2**		**MCM3**		**MCM4**		**MCM5**	
		**Cor**	***P***	**Cor**	***P***	**Cor**	***P***	**Cor**	***P***	**Cor**	***P***
CD8+ T cell	CD8A	-0.181	**0.000**	0.023	0.600	0.033	0.466	-0.072	0.106	-0.071	0.111
	CD8B	-0.150	**0.001**	0.132	**0.003**	0.088	**0.049**	-0.030	0.510	-0.022	0.617
	GZMA	-0.279	**0.000**	-0.058	0.197	-0.056	0.207	-0.125	**0.005**	-0.149	**0.001**
B cell	CD19	-0.051	0.259	-0.094	**0.036**	-0.051	0.257	-0.117	**0.009**	-0.056	0.209
	CD79A	-0.065	0.148	-0.051	0.254	-0.089	**0.045**	-0.115	**0.010**	-0.054	0.228
	MS4A1	-0.095	**0.034**	-0.074	0.096	-0.067	0.133	-0.143	**0.001**	-0.090	**0.044**
T cell	CD3D	-0.230	**0.000**	-0.093	**0.037**	-0.083	0.063	-0.183	**0.000**	-0.142	**0.001**
	CD3E	-0.157	**0.000**	-0.057	0.203	-0.041	0.359	-0.123	**0.006**	-0.081	0.070
	CD2	-0.191	**0.000**	-0.046	0.301	-0.044	0.330	-0.136	**0.002**	-0.097	**0.030**
TAM	CCL2	-0.028	0.529	-0.089	**0.046**	-0.119	**0.008**	-0.125	**0.005**	-0.105	**0.019**
	CD68	-0.085	0.057	-0.130	**0.004**	-0.204	**0.000**	-0.087	0.052	-0.137	**0.002**
	IL10	-0.121	**0.007**	-0.106	**0.018**	-0.192	**0.000**	-0.166	**0.000**	-0.150	**0.001**
M1	IRF5	0.038	0.402	-0.062	0.166	-0.007	0.876	-0.101	**0.024**	0.043	0.340
	PTGS2	0.153	**0.001**	-0.085	0.058	-0.127	**0.004**	-0.014	0.758	-0.103	**0.021**
	NOS2	0.157	**0.000**	0.226	**0.000**	0.193	**0.000**	0.218	**0.000**	0.230	**0.000**
M2	MS4A4A	-0.194	**0.000**	-0.137	**0.002**	-0.214	**0.000**	-0.200	**0.000**	-0.191	**0.000**
	CD163	0.323	**0.004**	0.075	0.510	-0.005	0.965	-0.107	0.347	-0.006	0.959
	VSIG4	0.243	**0.031**	0.018	0.877	-0.016	0.888	-0.165	0.147	-0.039	0.734
Neutrophils	ITGAM	0.227	**0.045**	0.004	0.974	-0.068	0.550	-0.168	0.140	-0.067	0.558
	CCR7	0.071	0.536	-0.074	0.516	-0.161	0.157	-0.118	0.299	-0.125	0.273
	SIGLEC5	0.296	**0.008**	0.215	0.057	0.193	0.089	0.161	0.156	0.168	0.140
DC	HLA-DQB1	0.105	0.357	-0.073	0.522	-0.222	**0.049**	-0.234	**0.038**	-0.108	0.344
	HLA-DRA	0.163	0.151	-0.111	0.328	-0.154	0.176	-0.228	**0.044**	-0.170	0.133
	HLA-DPA1	0.091	0.426	-0.128	0.259	-0.207	0.067	-0.242	**0.032**	-0.182	0.109
	CD1C	0.107	0.350	-0.138	0.225	-0.158	0.164	-0.162	0.154	-0.230	**0.041**
	NRP1	0.487	**0.000**	0.419	**0.000**	0.486	**0.000**	0.420	**0.000**	0.506	**0.000**
Th1	TBX21	-0.023	0.837	-0.071	0.532	-0.212	0.060	-0.193	0.088	-0.133	0.244
	STAT1	0.337	**0.003**	0.419	**0.000**	0.424	**0.000**	0.438	**0.000**	0.414	**0.000**
Th2	STAT6	0.181	0.110	-0.070	0.541	-0.036	0.754	0.185	0.102	-0.207	0.067
	GATA3	0.104	0.360	0.390	**0.000**	0.344	**0.002**	0.318	**0.004**	0.441	**0.000**
	STAT5A	0.204	0.072	0.030	0.791	-0.164	0.150	-0.038	0.736	-0.206	0.069
	IL13	0.074	0.518	0.090	0.432	0.190	0.093	0.151	0.183	0.200	0.078
Tfh	BCL6	0.417	**0.000**	0.212	0.061	0.319	**0.004**	0.303	**0.007**	0.264	**0.019**
Th17	STAT3	0.570	**0.000**	0.416	**0.000**	0.364	**0.001**	0.425	**0.000**	0.275	**0.015**
	IL17A	0.198	0.081	0.112	0.325	0.096	0.401	0.122	0.283	0.185	0.103
Treg	FOXP3	0.004	0.975	0.236	**0.037**	0.107	0.346	0.151	0.185	0.268	**0.017**
	STAT5B	0.387	**0.000**	0.154	0.175	0.123	0.279	0.239	**0.034**	0.011	0.922
	CCR8	0.021	0.855	0.191	0.091	-0.019	0.866	0.059	0.606	0.082	0.475
	TGFB1	0.184	0.104	0.315	**0.005**	0.196	0.083	0.228	**0.043**	0.255	**0.023**
CD8+ T cell	CD8A	0.050	0.261	-0.039	0.380	-0.102	**0.022**	-0.023	0.608	-0.035	0.440
	CD8B	0.073	0.102	0.033	0.464	-0.067	0.132	0.083	0.063	-0.002	0.961
	GZMA	-0.010	0.827	-0.097	**0.031**	-0.158	**0.000**	-0.118	**0.008**	-0.055	0.215
B cell	CD19	-0.106	**0.018**	-0.156	**0.000**	-0.107	**0.016**	-0.028	0.538	-0.169	**0.000**
	CD79A	-0.092	**0.040**	-0.169	**0.000**	-0.135	**0.002**	-0.078	0.081	-0.187	**0.000**
	MS4A1	-0.102	**0.023**	-0.153	**0.001**	-0.117	**0.009**	0.019	0.668	-0.160	**0.000**
T cell	CD3D	-0.088	**0.049**	-0.137	**0.002**	-0.202	**0.000**	-0.106	**0.017**	-0.137	**0.002**
	CD3E	-0.058	0.199	-0.123	**0.006**	-0.166	**0.000**	-0.078	0.082	-0.126	**0.005**
	CD2	-0.034	0.450	-0.113	**0.012**	-0.167	**0.000**	-0.056	0.211	-0.105	**0.019**
TAM	CCL2	-0.034	0.449	-0.151	**0.001**	-0.140	**0.002**	-0.061	0.170	-0.094	**0.035**
	CD68	-0.085	0.058	-0.185	**0.000**	-0.168	**0.000**	-0.119	**0.007**	-0.196	**0.000**
	IL10	-0.101	**0.024**	-0.215	**0.000**	-0.168	**0.000**	-0.069	0.123	-0.175	**0.000**
M1	IRF5	-0.068	0.129	-0.012	0.781	0.074	0.098	0.091	**0.041**	-0.110	**0.014**
	PTGS2	-0.100	**0.025**	-0.169	**0.000**	-0.051	0.253	-0.054	0.224	-0.049	0.276
	NOS2	0.148	**0.001**	0.148	**0.001**	0.206	**0.000**	0.201	**0.000**	0.175	**0.000**
M2	MS4A4A	-0.101	**0.024**	-0.248	**0.000**	-0.189	**0.000**	-0.085	0.056	-0.216	**0.000**
	CD163	-0.093	0.412	0.052	0.648	-0.073	0.521	0.147	0.198	-0.155	0.172
	VSIG4	-0.124	0.275	-0.010	0.931	-0.105	0.354	0.088	0.439	-0.225	**0.047**
Neutrophils	ITGAM	-0.163	0.151	-0.057	0.616	-0.078	0.493	0.201	0.076	-0.286	**0.011**
	CCR7	-0.150	0.186	-0.096	0.401	-0.062	0.586	0.030	0.790	-0.241	**0.033**
	SIGLEC5	0.079	0.488	0.215	0.057	0.136	0.231	0.105	0.359	0.076	0.503
DC	HLA-DQB1	-0.197	0.082	-0.107	0.346	-0.103	0.363	-0.036	0.755	-0.249	**0.027**
	HLA-DPB1	-0.161	0.157	-0.071	0.534	-0.082	0.473	0.089	0.437	-0.328	**0.003**
	HLA-DRA	-0.198	0.081	-0.154	0.176	-0.126	0.266	0.068	0.549	-0.375	**0.001**
	HLA-DPA1	-0.250	**0.027**	-0.190	0.093	-0.156	0.170	0.099	0.387	-0.417	**0.000**
	CD1C	-0.205	0.070	-0.251	**0.026**	-0.033	0.776	0.152	0.182	-0.364	**0.001**
	NRP1	0.402	**0.000**	0.546	**0.000**	0.341	**0.002**	0.202	0.074	0.451	**0.000**
Th1	TBX21	-0.201	0.076	-0.148	0.193	-0.115	0.313	0.076	0.505	-0.279	**0.013**
	STAT1	0.498	**0.000**	0.395	**0.000**	0.412	**0.000**	0.109	0.338	0.350	**0.002**
Th2	STAT6	-0.082	0.473	-0.069	0.544	0.284	**0.012**	0.419	**0.000**	-0.120	0.291
	GATA3	0.377	**0.001**	0.355	**0.001**	0.242	**0.032**	-0.125	0.271	0.406	**0.000**
	STAT5A	-0.144	0.205	-0.114	0.317	0.224	**0.048**	0.293	**0.009**	-0.264	**0.019**
	IL13	0.192	0.090	0.266	**0.018**	0.109	0.337	-0.036	0.752	0.361	**0.001**
Tfh	BCL6	0.229	**0.043**	0.336	**0.003**	0.271	**0.016**	0.237	**0.036**	0.234	**0.038**
Th17	STAT3	0.337	**0.003**	0.293	**0.009**	0.267	**0.018**	0.271	**0.016**	0.166	0.144
	IL17A	0.098	0.389	0.131	0.250	0.075	0.513	0.039	0.732	0.082	0.473
Treg	FOXP3	0.086	0.449	0.203	0.073	0.094	0.407	0.042	0.711	0.200	0.077
	STAT5B	0.100	0.382	0.121	0.289	0.254	**0.024**	0.280	**0.012**	-0.108	0.345
	CCR8	-0.012	0.920	0.046	0.688	0.190	0.094	0.260	**0.021**	-0.005	0.963
	TGFB1	0.234	**0.038**	0.196	0.083	0.214	0.058	0.077	0.500	0.184	0.104

Bold font indicates significant difference.

## DISCUSSION

The MCM family are ubiquitously expressed proteins that are involved in the initiation and progression of eukaryotic genome replication [17]. Numerous studies have reported aberrant expression of MCM family members in diverse tumors types, indicating their vital roles in tumorigenesis and cancer progression [18–20]. However, the function of MCM family members in LUSC has not been systematically examined. Hence, we explored the differential mRNA expression of each MCM family member in LUSC tissues compared to in normal tissues. MCM2–10 mRNAs were overexpressed in LUSC cells compared to in normal cells, suggesting their potential to act as oncogenes. Thus, the protein levels of all MCM members were upregulated in patients with LUSC.

Furthermore, numerous studies have been performed to investigate the relationship between clinicopathological features of patients with cancer and expression of MCMs [19, 21–23]. The association of the expression of MCMs with prognostic importance of cancer has also been extensively evaluated. Liu et al. found that MCM4/5/8 were significantly associated with worse OS of patients with lung adenocarcinoma and may serve as potential prognostic indicators for this disease [18]. Gou et al. suggested that high expression of some MCM members can serve as predictive biomarkers for poor prognosis in cancer [24]. Hence, we investigated the clinical correlation and prognostic relevance of abnormally expressed MCMs in patients with LUSC. The mRNA expression of MCM family members in LUSC tissues were significantly correlated with lymph node metastasis and clinicopathological stages. Our results also suggested that MCM3 and MCM7 were associated with better OS but poorer RFS and PPS in patients with LUSC. Overexpression of MCM4/5/6/8 was significantly associated with better OS, whereas that of MCM8 and MCM10 was associated with poorer RFS in patients with LUSC. Additionally, overexpression of the MCM1/2/4/5/9 mRNAs was associated with poor PPS. Further, we found a correlation between the expression of MCMs and prognostic value of the MCM family at different clinical stages of LUSC. MCM2/3/4/5/8/9 expression were significantly associated with poorer RFS in patients with stage III LUSC. Thus, this gene family may have important prognostic value, which should be further evaluated.

Genetic alterations are common in various tumors, including LUSC, and play critical roles in several biological processes such as cell growth, apoptosis, and the cell cycle. Methylation is one genetic alteration with an important role in cancer development. DNA methylation primarily suppresses gene expression, although it can sometimes promote gene expression, according to a previous review [25]. Yin et al. also identified numerous transcription factors that preferentially bind to CpG-methylated sequences [26]. We found that MCM genes were abnormally expressed in LUSC tissues, which was likely related to DNA methylation. This result indicates that DNA methylation-targeted drugs can be used to treat cancer. Essential DNA replication factors, which are highly expressed in malignant cancer cells and precancerous cells but downregulated in differentiated somatic cells, make MCM proteins good targets for anti-cancer drugs. Various small-molecule MCM-targeting inhibitors were recently identified as an initial step toward therapeutic development [27, 28]. Mutation analysis revealed several genetic alterations in all members of the MCM family in patients with LUSC, with 45% of genes altered in LUSC tissues, leading to upregulated and altered mRNA transcription. We also investigated the co-expression of different molecules, including CNTD1, TAS2R5, and LY6G5B, with MCMs. The most significant pathways in which MCMs were involved were cytokine–cytokine receptor interaction and antigen processing and presentation. Therefore, the MCM family shows potential as therapeutic targets for LUSC through interactions with key molecules in immune infiltration-related pathways in tumors.

Previous studies showed that immune cell infiltration affects tumor progression and recurrence while also playing an important role in determining the response to immunotherapy and clinical outcomes [29–31]. LUSC is a genetically complex and heterogeneous disorder with no effective therapies. The tumor immune response was recently shown to be essential for the genesis and development of LUSC [32, 33]. Given that the human immune system is critical in the onset and development of LUSC [34, 35], identifying the genes associated with immune cells in LUSC could be beneficial. Nevertheless, the relationships between MCM proteins and immunotherapy of LUSC have not been reported. Our results showed that all members of the MCM family are related to six types of immune cells, including B cells, CD4+ T cells, CD8+ T cells, macrophages, neutrophils, and dendritic cells. Therefore, we investigated the association between the mRNA expression of MCM family members and markers of immune infiltration in patients with LUSC. Contrary to expectations, several immune cells showed strong associations with the expression of MCM family members. Thus, MCM family members may be crucial in the development of the localized TME in LUSC tissues and can be used as immunotherapeutic targets for LUSC. There was limitation in the present study. Most data utilized in this study were acquired from online databases. To corroborate our findings, further cell-based research and clinical experiments in a well-established tumor cohort are essential.

## CONCLUSIONS

In conclusion, we comprehensively analyzed the expression of the MCM family in patients with LUSC and the relationship between MCM expression and the prognosis of these patients using bioinformatic tools. Our results improve the understanding of the vital role of MCMs in tumor progression and the immune response in patients with LUSC. The MCM family may be useful as biomarkers and therapeutic targets and can be used to develop diagnostic and prognostic approaches to improve treatment outcomes.

## MATERIALS AND METHODS

### TIMER

Based on The Cancer Genome Atlas database, the TIMER tool is a comprehensive resource for evaluating immune cell infiltration and the clinical outcomes of 10,897 tumors from 32 different cancer types. This database enables analyses of the correlations between genes and immune-infiltrating cells, comparisons of gene expression in tumors and normal tissues in various malignancies, and survival analysis, among other functions [36]. We investigated the mRNA expression of MCM family members in various malignancies or specific cancer subtypes from TIMER, which contains 501 LUSC samples and 51 normal lung samples; the log2 (transcripts per million) were applied to convert the data to a log-scale. We also examined the relationship between the MCM family and immune cell infiltration using the TIMER database.

### GEPIA2

The web-based tool GEPIA2 provides vital interactive and customizable functions, including patient survival analysis, correlation analysis, and differential expression analysis [37]. 338 normal lung samples and 486 primary LUSC samples were included in the GEPIA2 dataset. The GEPIA2 datasets were utilized to compare the expression of different MCMs in LUSC and normal tissues, and *p* < 0.05 was considered to indicate statistically significant results.

### Human protein atlas

Human Protein Atlas is an online database containing information on protein expression in various cancer types based on immunohistochemistry [38]. We used immunohistochemical images to evaluate the protein levels of several MCM members between LUSC tumors and normal lung tissues.

### UALCAN

UALCAN is an interactive network resource based on The Cancer Genome Atlas datasets that allows users to compare information on DNA methylation and mRNA expression in human malignancies [39]. To examine the relationships between the mRNA expression of various MCMs and clinicopathological features in 856 LUSC samples and 52 normal lung samples, we used the “individual cancer stages” model and “nodal metastatic status” model. Using the UALCAN database, we further predicted DNA methylation alterations in MCM family members from 370 LUSC cases and 42 normal lung tissues. Student’s *t*-test was applied, and *p* < 0.05 was considered to indicate statistically significant results.

### Kaplan–Meier plotter database

The Kaplan–Meier plotter database (https://kmplot.com) was used to analyze the prognostic value of the MCM family in LUSC. The mRNA expression value of the auto-selected best cutoff was used to split the LUSC samples into high and low expression groups to investigate the relationship between MCM expression and OS, RFS, and PPS for 501 patients with LUSC. Using the Kaplan–Meier plotter database, we further examined the prognostic value of MCM family members in patients with various clinical stages of LUSC. A *p*-value less than 0.05 was considered to indicate statistically significant results.

### cBioPortal

cBioPortal (http://cbioportal.org), an open comprehensive platform, can be used to analyze multidimensional cancer genomics and clinical data. We examined the genomic map of the MCM family members, which contains information on mutations and mRNA expression, in 178 LUSC samples. The threshold of the |log2 fold-change| was 0.45, and the *p*-value cutoff was set at 0.05.

### STRING

The STRING database (https://cn.string-db.org/) was used to assess correlations involving MCM genes.

### Cytoscape

We integrated functionally 186 co-expressed MCM family members selected using the cBioPortal (molecule names are provided in Supplementary Table 1). The node size was determined as the degree values between interacting proteins.

### Metascape

The web-based analysis toolkit Metascape is comprehensive, efficient, customizable, and interactive [40]. Using this dataset, we conducted GO and KEGG pathway enrichment analyses for genes co-expressing with MCM members.

### Data availability statement

The original contributions presented in the study are included in the Supplementary Materials, further inquiries can be directed to the corresponding authors.

## Supplementary Material

Supplementary Table 1
